# Unveiling the Hidden Burden: A Systematic Review on the Prevalence and Clinical Implications of Calcified Brain Metastases

**DOI:** 10.3390/biom14121585

**Published:** 2024-12-11

**Authors:** Alexandru Garaba, Pier Paolo Panciani, Aida Da’ana, Tamara Ius, Alessandro Tel, Marco Maria Fontanella, Marco Zeppieri, Fulvia Ortolani, Edoardo Agosti

**Affiliations:** 1 Department of Surgical Specialties, Radiological Sciences and Public Health, University of Brescia, 25123 Brescia, Italy; alexgarabaflb@gmail.com (A.G.); edoardo_agosti@libero.it (E.A.);; 2 Medical School Faculty, Al-Quds University, Abu Dis P.O. Box 19356, East Jerusalem, Palestine; 3 Neurosurgery Unit, Head-Neck and NeuroScience Department, University Hospital of Udine, p.le S. Maria Della Misericordia 15, 33100 Udine, Italy; 4 Clinic of Maxillofacial Surgery, Head-Neck and NeuroScience Department University Hospital of Udine, p.le S. Maria Della Misericordia 15, 33100 Udine, Italy; 5 Department of Ophthalmology, University Hospital of Udine, Piazzale S. Maria Della Misericordia 15, 33100 Udine, Italy; 6 Department of Medicine, University of Udine, 33100 Udine, Italy

**Keywords:** brain metastasis, calcification, computed tomography, systematic review

## Abstract

Background: Brain calcifications, found in various conditions, may be incidental or crucial for diagnosis. They occur in physiological changes, infections, genetic diseases, neurodegenerative conditions, vascular syndromes, metabolic disorders, endocrine disorders, and primary tumors like oligodendroglioma. While often incidental, their presence can be vital for accurate diagnosis. Brain metastases are the most common neoplastic lesions in adults, with their incidence increasing due to improved diagnostic tools and overall oncologic patient survival. Calcifications within brain metastases are uncommon, mostly seen in patients treated with radiation therapy (RT). Although cases of calcified brain metastasis (CBM) are reported, large recent studies are scarce and the real incidence remains unclear. This lack of data raises the risk of underestimating CBM in the differential diagnosis of brain calcifications, potentially leading to misdiagnosis and delayed treatment, particularly when calcifications are observed without prior RT. Aim: This systematic review sought to assess the incidence of CBM in patients with identified primary tumors who underwent brain chemotherapy (CT) for staging. Additionally, the study aimed to explore the primary tumor types more frequently linked to CBM and determine whether CBM manifested initially or post-RT. Methods: A comprehensive search was performed across prominent medical databases (PubMed, Cochrane Library, and Embase) until 20 January 2024. The employed search method incorporated pertinent Medical Subject Headings (MeSH) and keywords such as “calcification”, “brain metastasis”, and “CT scan”. Studies included in this review were publications focusing on CBM in patients with identified primary tumors who underwent brain CT for staging. Results: In a systematic review of 39 studies on CBM in patients with identified primary tumors, 98 papers were initially identified, with 52 chosen for full-text analysis. Among them, 39 were deemed eligible after excluding 13 for various reasons. The study investigates brain calcifications in 1115 patients with metastatic disease, revealing that 7.89% had brain metastases, with 25% showing calcifications ab initio. These calcifications were more common than previously reported, emphasizing the need for attention to intraparenchymal brain calcifications in oncologic patients. Most CBM originated from lung and breast adenocarcinomas, and their correlation with primary tumor calcifications was inconclusive. Conclusions: The study highlights the significance of identifying evolving lesions in oncologic patients, calling for increased awareness among neuroradiologists and shedding light on the prevalence and characteristics of CBM.

## 1. Introduction

Brain calcifications are frequently encountered in a wide variety of conditions, ranging from physiological aging to infections, genetic and neurodegenerative disorders, vascular abnormalities, metabolic conditions, and various neoplasms. While calcifications are often considered incidental findings, their detection can be critical in guiding differential diagnoses [1].

Brain metastases represent the most common type of brain neoplasm in adults, and their incidence has increased over the years. This rise is attributed to the improved survival rates of cancer patients, alongside advancements in imaging techniques such as high-resolution computed tomography (CT) and magnetic resonance imaging (MRI), which have greatly enhanced our ability to detect brain metastases [2]. However, the occurrence of calcifications within brain metastases remains a relatively rare phenomenon occurring in a small percentage of cases, with reported incidences of 1.1% in surgical specimens and 6.6% in autopsies [3]. It appears to be most frequently associated with patients who have undergone brain radiotherapy (RT) [4]. Despite several reported cases of calcified brain metastases (CBM), large-scale studies investigating the true incidence and characteristics of this condition remain scarce, leaving many aspects of CBM poorly defined.

The presence of calcifications in brain metastases may be underestimated, especially in cases where patients have not received prior RT. This poses a significant risk of misdiagnosis or delayed treatment, particularly when calcifications are observed in the early stages of disease progression. CBM can easily be mistaken for other forms of brain calcification, which can complicate treatment strategies and prognosis if not correctly identified.

Several primary tumors have been associated with the development of CBM, including lung cancers, breast adenocarcinoma, sarcomas, non-Hodgkin’s lymphoma, and gastrointestinal cancers such as colorectal and pancreatic adenocarcinomas [5,6,7,8,9,10]. Identifying these associations is crucial for understanding the clinical course of CBM and guiding personalized treatment strategies.

This review presents the inaugural comprehensive investigation of CBM, a rare and inadequately investigated occurrence in oncology patients. In contrast to earlier research that mostly concentrates on individual case reports or a limited series of case reports, this systematic review summarizes the literature data, elucidating the prevalence, primary tumor relationships, and clinical implications of CBM. Moreover, this study aims to highlight the occurrence of calcifications at initial diagnosis, irrespective of previous radiation, providing essential insights into their diagnostic and prognostic significance. By providing a deeper understanding of CBM, this review hopes to shed light on its clinical implications, thereby improving diagnostic accuracy and informing treatment decisions.

## 2. Materials and Methods

### 2.1. Literature Review

The systematic review was conducted in accordance with the Preferred Reporting Items for Systematic Reviews and Meta-Analyses (PRISMA) guidelines and statement [11]. The study review was not registered. Two writers conducted a thorough and comprehensive literature search throughout the PubMed, Ovid MEDLINE, and Ovid EMBASE databases. The initial literature search was conducted on 10 August 2024, and the search was subsequently updated on 9 September 2024. A search strategy was developed through a combination of keyword searches. The search terms, comprising “brain metastases”, “cerebral metastases”, “calcification”, “calcified”, “computed tomography”, and “CT scan”, were employed in both AND and OR combinations. Studies were obtained utilizing the subsequent Medical Subject Headings (MeSH) phrases and Boolean operators: (“brain metastasis” OR “brain metastases” OR “cerebral metastasis” OR “cerebral metastases”) AND (“calcification” OR “calcified”) AND (“CT scan” OR “computed tomography” OR “CT”).

Further pertinent articles were discovered by cross-referencing the bibliographies of chosen studies. A search filter was established to display only papers within the specified timeframe: 1980–2024. All studies were chosen according to the subsequent inclusion criteria: (1) case reports, case series, and retrospective studies documenting instances of CBM; and (2) studies involving patients with a verified diagnosis of primary cancers who underwent brain imaging for staging purposes. The subsequent exclusion criteria were applied: (1) reviews, meta-analyses, editorials, and letters to the editor; (2) studies lacking clear definitions of methodology and/or outcomes; and (3) studies addressing brain calcifications not associated with metastases. The compilation of recognized studies was imported into Endnote X9, and duplicates were eliminated. Two separate researchers (E.A. and P.P.P.) verified the results based on the inclusion and exclusion criteria. A third reviewer (M.Z.) adjudicated all discrepancies. The qualifying articles underwent comprehensive full-text evaluation.

### 2.2. Data Extraction

For each study included in this review, we extracted the following information: authors, year and journal of publication, study design, sample size, primary tumor type, imaging modality used, location and type of brain calcifications, whether the patient received brain radiotherapy, and survival rates.

### 2.3. Outcomes

The main outcomes encompassed the incidence of CBM in patients with identified primary tumors. Secondary outcomes included the characteristics of CBM in relation to different primary tumors, whether these calcifications were present at the initial diagnosis or developed subsequent to RT, and the relation between CBM and overall survival.

### 2.4. Risk of Bias Assessment

**Figure A1:**
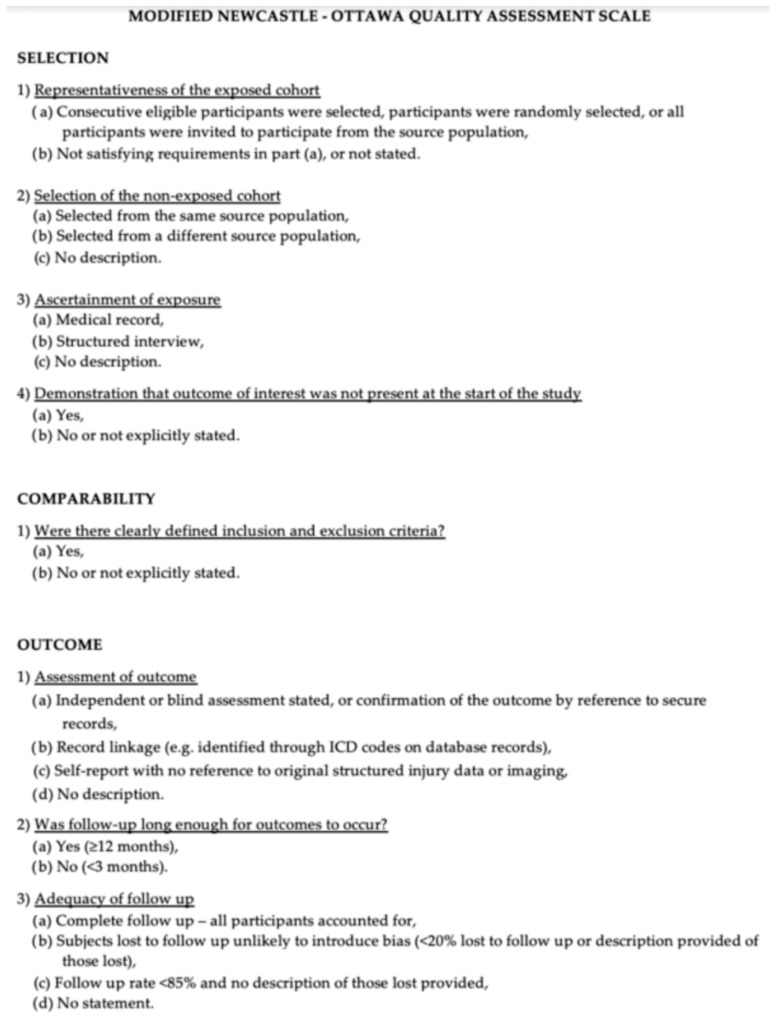
Modified Newcastle–Ottawa This scale is utilized for evaluating the quality of observational studies, and assesses selection criteria, comparability, and outcome evaluation. A higher score signifies enhanced study quality.

The Newcastle–Ottawa Scale (NOS) was employed to assess the quality of the included observational studies by evaluating selection criteria, comparability, and outcome assessment. Case reports and series were evaluated utilizing the Joanna Briggs Institute (JBI) Critical Appraisal Checklist, which assesses essential elements like the clarity of case descriptions, intervention specifics, and clinical results. Two separate evaluators conducted the assessments, with discrepancies adjudicated by a third evaluator. Reports that received seven or more points were deemed high-quality studies. The Newcastle–Ottawa Scale (NOS) [12] was employed to evaluate the quality of the included observational reports. A quality assessment was conducted by evaluating the selection criteria, study comparability, and outcome evaluation. The optimal score was 9. Elevated ratings signified superior study quality. Reports that received seven or more points were classified as high-quality studies. For case reports and case series, we used the Joanna Briggs Institute (JBI) Critical Appraisal Checklist to assess study quality. The JBI tool evaluates key aspects such as the clarity of the case description, patient history, interventions, and clinical outcomes. Each study was assessed for the presence of bias and the quality of the reporting. The 2 authors (P.P.P. and E.A.) provided the quality assessment independently. When there were discrepancies, the third author was invited to review assess the paper (Figure A1).

### 2.5. Statistical Analysis

Descriptive statistics including percentages and ranges were included. The analyses were completed using the R statistical v3.4.1 http://www.r-project.org (accessed on 12 September 2024).

## 3. Results

### 3.1. Literature Review Results

**Figure 1:**
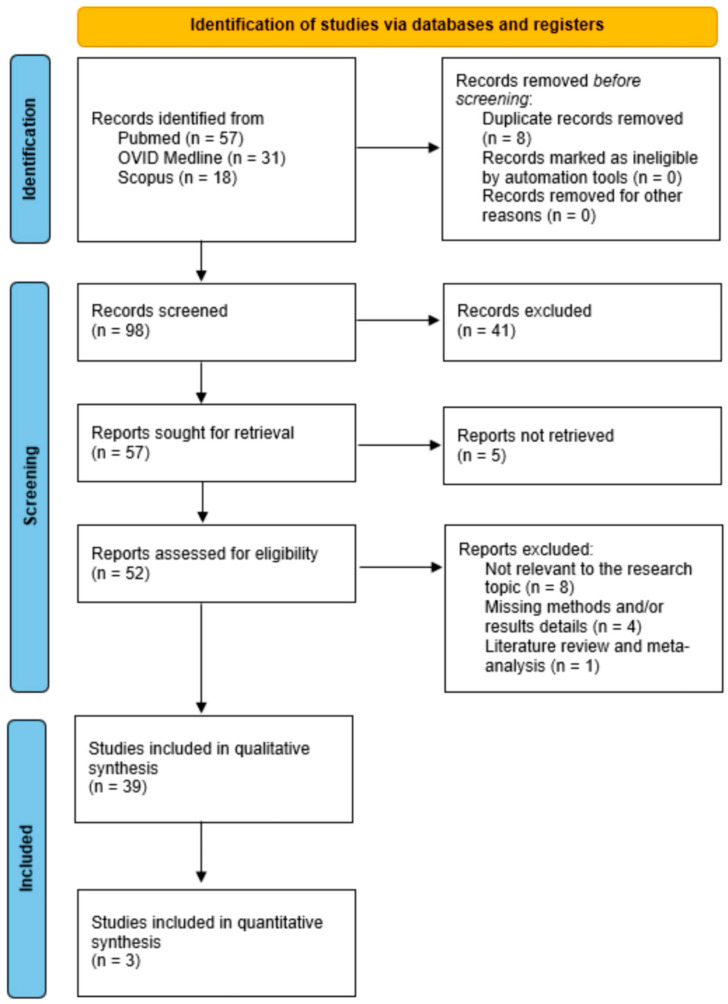
PRISMA flowchart.

A total of 98 papers were identified following the removal of duplicates. Following the analysis of titles and abstracts, 57 articles were selected for comprehensive review. Eligibility was determined for 52 articles. Thirteen articles were excluded for the following reasons: (1) irrelevance to the research topic (eight articles), (2) absence of methods and/or results details (four articles), and (3) classification as literature review and meta-analysis (one article). All studies incorporated in the analysis possessed at least one outcome measure for one or more of the patient groups examined. Figure 1 illustrates the flowchart in accordance with the PRISMA statement.

**Figure A2:**
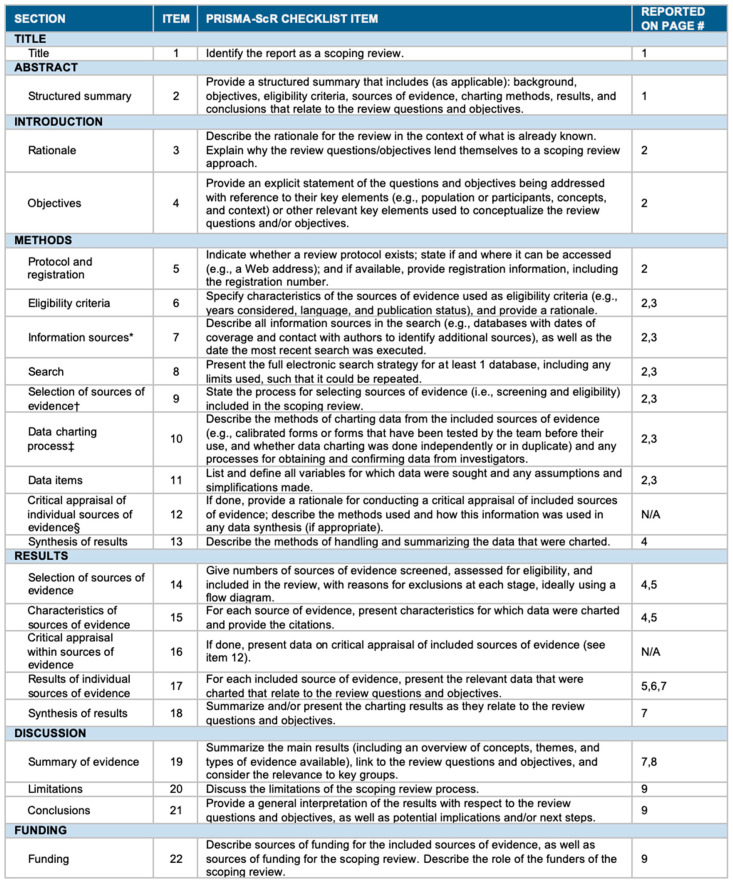
PRISMA-ScR checklist (Preferred Reporting Items for Systematic Reviews and Meta-Analyses extension for Scoping Review). JBI = Joanna Briggs Institute; PRISMA-ScR = Preferred Reporting Items for Systematic reviews and Meta-Analyses extension for Scoping Reviews. * Where sources of evidence (see second footnote) are compiled from, such as bibliographic databases, social media platforms, and Web sites. † A more inclusive/heterogeneous term used to account for the different types of evidence or data sources (e.g., quantitative and/or qualitative research, expert opinion, and policy documents) that may be eligible in a scoping review as opposed to only studies. This is not to be confused with information sources (see first footnote). ‡ The frameworks by Arksey and O’Malley (6) and Levac and colleagues (7) and the JBI guidance (4, 5) refer to the process of data extraction in a scoping review as data charting. § The process of systematically examining research evidence to assess its validity, results, and relevance before using it to inform a decision. This term is used for items 12 and 19 instead of “risk of bias” (which is more applicable to systematic reviews of interventions) to include and acknowledge the various sources of evidence that may be used in a scoping review (e.g., quantitative and/or qualitative research, expert opinion, and policy document) [47].

The PRISMA checklist can be found in Appendix A (Figure A2).

### 3.2. Data Analysis

A summary of the included reports is presented in Table 1, Table 2 and Table 3.

A total of 39 studies were included in the qualitative analysis, comprising 33 case reports, three case series, and three observational reports. The case reports and case series provided detailed information on primary tumor types, locations, and characteristics of CBM, as well as clinical outcomes. The primary tumors most frequently associated with CBM were lung adenocarcinoma and breast carcinoma, observed in 44.4% (16/36) and 16.6% (6/36) of the studies, respectively. Osteosarcoma was the next most common, appearing in 13.9% (5/36) of the reports. Calcifications were primarily located in the cerebellum and frontal lobes, followed by the parietal and occipital lobes. The most common calcification types were punctate and nodular. In terms of clinical outcomes, survival times varied widely, ranging from just a few days to three years and three months. Five reports (13.9%) documented calcifications that appeared after RT, suggesting a potential link between radiotherapy (RT) and the development of CBM.

Quantitative analysis was conducted based on the three observational reports included in the review. These studies provided numerical data on the prevalence of CBM, with rates ranging from 5.2% to 26.3% in patients with brain metastases from various primary tumors, including lung adenocarcinoma, breast cancer, and ovarian carcinoma. The pooled prevalence of CBM across these reports was 7.89%. The median overall survival (OS) for patients with CBM was reported in Study 1 as 462 days (approximately 15.4 months). Unfortunately, survival data were not available from the other observational studies, which limited the ability to perform a more comprehensive comparative survival analysis. The impact of radiotherapy on the development of calcifications and its influence on survival outcomes were analyzed, but the results were mixed, with small sample sizes preventing definitive conclusions.

## 4. Discussion

This review provides an in-depth analysis of CBM, offering new insights into the qualitative and quantitative findings from case reports, case series, and observational studies. The results of this review indicate that lung adenocarcinoma and breast carcinoma are the most frequent primary tumors associated with CBM. This aligns with the existing literature, which has consistently identified lung and breast cancers as the most common sources of brain metastases [45]. However, the formation of calcifications in metastases is insufficiently investigated. In addition, osteosarcoma was identified as another frequent cause of calcified brain metastases, which is consistent with its known tendency to calcify in metastatic lesions [46]. These findings suggest that CBM might be more common than previously recognized, especially given the pooled prevalence of 7.89% reported across the observational studies. This prevalence is higher than expected and highlights the need for increased clinical awareness. This raises important questions about the underlying pathophysiological mechanisms of calcification in brain metastases, and whether it is a tumor-specific phenomenon or related to prior treatment or other factors.

The data on clinical outcomes, particularly survival, were limited by the fact that only one study provided OS data (462 days) [43]. The wide variation in survival times observed in case reports, which ranged from a few days to over three years, highlights the heterogeneity of this patient population. Although some studies suggested a potential link between RT and calcifications, with 13.9% of cases documenting calcifications post-RT, the overall impact of radiotherapy on CBM development remains uncertain.

This review’s findings highlight the complex characteristics of CBM, stressing the significance of early detection and accurate classification in clinical practice. A notable observation is the high incidence of CBM in patients with lung adenocarcinoma and breast carcinoma, consistent with current evidence that designates these malignancies as primary contributors to brain metastases. The reported pooled prevalence of 7.89% contests previously understated statistics and indicates that CBM may not be as uncommon as originally believed. This acknowledgment necessitates a reevaluation of diagnostic techniques, especially in instances of detected calcifications absent prior RT, requiring a comprehensive differential diagnosis to exclude alternative causes. The variety in survival outcomes highlights another significant facet of CBM, illustrating differences in tumor biology, treatment responses, and patient demographics. The solitary observational study indicating a median overall survival (OS) of 462 days serves as a reference point; nonetheless, the significant variability in survival durations, spanning from days to years, underscores the necessity for personalized patient therapy. The role of radiotherapy (RT) in the production of calcified bone metastases (CBM) is unclear, requiring further research to determine if calcifications result directly from RT, are an intrinsic feature of specific malignancies, or arise from a mix of both factors. This ambiguity highlights the potential for calcifications to act as a prognosis indicator or to affect therapeutic decision-making.

The finding of CBM highlights the importance of sophisticated imaging modalities, such as high-resolution CT and MRI, in revealing complex features of cerebral lesions. The differentiation among punctate, nodular, and amorphous calcification types not only aids in identifying CBM but may also provide insights into the underlying pathophysiological processes. Punctate calcifications may signify a slow progression of tumor necrosis and calcification, whereas nodular patterns could suggest more aggressive or biologically different tumor subtypes. The spatial distribution of CBM, primarily in the cerebellum and frontal lobes, introduces further complication, indicating a possible predilection of metastatic cancers for particular brain regions. Comprehending these patterns may illuminate tumor biology and metastatic routes.

The clinical implications of CBM encompass not just diagnosis and prognosis but also therapy issues. Calcifications may affect surgical methods, as lesions with significant calcification could provide technical difficulties. Likewise, radiation and chemotherapy protocols may require modification to accommodate the distinct attributes of calcified metastases. The review’s conclusions advocate for an interdisciplinary strategy, integrating knowledge from neurology, cancer, radiology, and pathology to improve patient outcomes. Future research should emphasize longitudinal investigations with bigger cohorts to corroborate the preliminary findings of this analysis and explore the molecular mechanisms driving calcification in metastatic brain lesions. By addressing these gaps, the medical community can refine diagnostic accuracy, optimize treatment options, and ultimately, improve the quality of care for patients with CBM.

Although the reviewed studies did not incorporate direct imaging data of the mineral microstructure, the existing literature indicates that calcifications in CBM may display both amorphous and crystalline structures, with distribution patterns differing between intercellular and extracellular spaces. Research has recognized hydroxyapatite as a prevalent crystalline element in calcifications, in conjunction with amorphous deposits linked to degenerative phenomena. Future research employing improved imaging techniques, including electron microscopy and spectroscopy, is necessary to clarify the distribution of mineral particles concerning brain cell populations and the mechanisms underlying these processes.

The complex etiology of ectopic calcification in the brain, as emphasized in the literature, requires a multidisciplinary approach to tackle its pathogenesis and treatment methods. The convergence of genetics, molecular biology, and radiological imaging offers a promising avenue for future research to enhance the understanding of the biomineralization process. Incorporating knowledge from neuroscience, cancer, and materials science may expand therapy alternatives beyond surgical methods, providing focused strategies to diminish morbidity and mortality linked to calcifications.

Recognizing CBM on imaging could have important diagnostic and prognostic value, prompting clinicians to consider this in their differential diagnosis and treatment planning. Additionally, the presence of calcifications pre-RT suggests the possibility of the earlier identification of metastases with distinct radiological characteristics. The role of radiotherapy in CBM formation, although not conclusively established, warrants further investigation.

A limitation of this study is the inconsistent reporting of demographic and clinical details, such as race, age, and medical treatments, in the included literature. The absence of standardized reporting across studies made it challenging to synthesize these variables in a systematic manner or to assess their potential influence on the development of calcifications in brain metastases. As a result, our review may lack a comprehensive exploration of demographic and treatment-related factors that could provide further insights into patient-specific characteristics. Future research should aim to include these details consistently to facilitate more robust analyses and enhance our understanding of the underlying mechanisms and clinical implications.

## 5. Conclusions

This systematic review sheds light on the prevalence, characteristics, and potential clinical implications of CBM. The review found that CBM is frequently associated with primary tumors such as lung adenocarcinoma, breast carcinoma, and osteosarcoma. Although calcifications were historically linked to RT, the presence of CBM at initial diagnosis in a significant proportion of cases suggests that other biological mechanisms may be responsible.

Despite the pooled prevalence of 7.89% for CBM across observational studies, data on clinical outcomes were limited, and no firm conclusions could be drawn regarding the impact of CBM on overall survival. The findings suggest that further research is necessary to clarify the relationship between RT and CBM formation, explore the biological mechanisms underlying these calcifications, and determine the prognostic value of CBM in patients with metastatic disease.

Future studies should focus on larger cohorts and prospective designs to better assess the prevalence, clinical significance, and long-term outcomes of patients with CBM. A deeper understanding of CBM could improve diagnostic accuracy and potentially guide more personalized treatment strategies for patients with brain metastases.

## Figures and Tables

**Table 1:** Summary of the case reports included in this review.

Author	Year	Primary Tumor Type	Location of CBM	Type of CBM	Radiotherapy	Overall Survival (OS)
Tomita and Larsen [13]	1983	Metastatic undifferentiated sarcoma	Parietal/cerebellum	-	Yes	3 mo
Kelly et al. [14]	1987	Lung large cell undifferentiated carcinoma	Left cerebral hemisphere	-	No	9 mo
Fukuda et al. [15]	1988	Lung papillary adenocarcinoma	Disseminate	Nodular	No	-
Callizo et al. [10]	1989	Acinar cell carcinoma of the pancreas	Disseminate	Punctate/curvilinear comma-like scattered	Yes	-
Gaze et al. [16]	1989	Squamous carcinoma of cervix	Temporoparietal	Punctate	Yes	10 mo
Yamada and Suzuki [17]	1989	Diffuse, large cell non-Hodgkin’s lymphoma	Thalamic region	Nodular	No	12 mo
Tashiro et al. [3]	1990	Metastatic squamous cell carcinoma/unknown origin	Corpus callosum	Conglomerate	No	12 mo
Nakase et al. [18]	1991	Small cell carcinoma of lung	Disseminate	-	-	-
Kincaid [19]	1992	Osteosarcoma	Occipital lobe	-	No	8 mo
Yamazaki et al. [20]	1993	Metastatic adenocarcinoma of the lung	Disseminate	Scattered punctate	No	12 d
Bhatoe and Gill [8]	1994	Non-Hodgkin’s lymphoma	Frontal lobe	-	No	-
Ricke et al. [21]	1996	Papillary carcinoma of the ovaries	Disseminate	Circular/irregular	-	1 mo
Cacho et al. [22]	1996	Lung carcinoma	Right lateral ventricle, right paramedian region, left frontoparietal cortical region	-	-	12 mo
Duval et al. [23]	1996	Lung carcinoma	-	-	No	-
Stadnik et al. [24]	1997	Papillary cystadenocarcinoma	Posterior fossa	Peripheral calcification	-	-
Dibiane et al. [25]	1998	Bronchial adenocarcinoma	-	-	Yes	-
Henriquez et al. [26]	1999	Ovarian carcinoma	-	-	No	20 d
Teksam et al. [27]	2004	Osteosarcoma	Temporoparieto-occipital	-	-	2 mo
Graña et al. [28]	2007	Adenocarcinoma of colorectal	Frontal lobe	-	-	24 mo
Pugnet et al. [29]	2007	Breast carcinoma	Supra/infratentorial	-	No	-
Fatehi et al. [6]	2010	Breast cancer	Disseminate	-	No	12 mo
Inomata et al. [5]	2012	Lung adenocarcinoma/leptomeningeal adenocarcinoma	Disseminate	Scattered	-	-
Eom and Kim [30]	2012	Non-small cell carcinoma of lung	Frontal lobe	-	No	-
Kawamura et al. [31]	2013	Serous mucinous adenocarcinoma	Cerebellar hemisphere	-	No	Few d
Fernandez et al. [32]	2013	Osteosarcoma of the mandible	Centrum semiovale	Round	No	-
Michail et al. [9]	2015	Adenocarcinoma of colorectal	Frontoparietal	-	Yes	-
Ressl and Oberdornfer [33]	2015	Breast invasive ductal carcinoma	-	-	No	-
Bahrami et al. [34]	2018	Adenocarcinoma, probably from colon origin	Parietal lobe	Amorphous	-	-
Vazquez et al. [35]	2020	Breast cancer	Disseminate	-	No	2 mo
Kokkali et al. [7]	2020	Osteosarcoma	Cerebellum	-	-	-
Ananthashayana et al. [36]	2020	Osteosarcoma	Left frontal lobe	-	-	3 mo
Divya et al. [37]	2022	Adenocarcinoma of lung	Parieto-occipital region	-	-	-
Mo et al. [38]	2023	Pulmonary adenocarcinoma	Left periventricular white matter, basal ganglia	-	-	-

Abbreviations: CBM = calcified brain metastasis; OS = overall survival; mo = months; d = days.

**Table 2:** Summary of the case series included in this review.

Author	Year	Primary Tumor Type	Location of CBM	Type of CBM	Radiotherapy	Overall Survival (OS)
Anand and Potts [39]	1982	Lung, breast	Temporal lobe	Punctate	No	-
		Lung	Supra/infratentorial	Punctate	No	-
		Esophagus	Cerebellum	Curvilinear	No	-
		Lung	Cerebellum	Curvilinear	No	-
		Colon	Frontal lobe	Amorphous	No	-
		Breast	Supratentorial	Punctate	No	-
		Colon	Frontoparietal	Amorphous	No	-
Hwang et al. [40]	1993	Metastatic adenocarcinoma	Parietal/temporal lobe, corpus callosum	-	No	27 mo
		Lung squamous cell carcinoma	Temporal lobe	-	No	45 mo
Ohmoto et al. [41]	2002	Lung adenocarcinoma	Frontal lobe	-	No	33 mo
		Adenocarcinoma	Frontal lobe	Linear	No	12 mo

Abbreviations: CBM = calcified brain metastasis; OS = overall survival; mo = months.

**Table 3:** Summary of the observational retrospective studies included in this review.

Author	Year	Primary Tumor Type	Sample Size	Patients with CBM	Incidence of CBM (%)	Radiotherapy	Overall Survival (OS)
Hwang et al. [42]	2016	Lung adenocarcinoma	19	5	26.3	No	-
Kuo et al. [43]	2017	NSCLC	943	49	5.2	No	462 d
Rebella et al. [44]	2020	Lung, breast, ovaries	153	34	22.2	16	-

Abbreviations: CBM = calcified brain metastasis; OS = overall survival; d = days; NSCLC = non squamous cell lung cancer.

## Data Availability

Data are available from a publicly accessible repository.

## Abbreviations

CT = computed tomography; MRI = magnetic resonance imaging; RT = radiotherapy; CBM = calcified brain metastases; PRISMA = Preferred Reporting Items for Systematic Reviews and Meta-Analysis; NOS = Newcastle–Ottawa scale; JBI = Joanna Briggs Institute.
